# Supplementary material for: Effect of Pit Mud on Bacterial Community and Aroma Components in Yellow Water and Their Changes during the Fermentation of Chinese Strong-Flavor Liquor
Source: Foods. 2020 Mar 23;9(3):372. doi: 10.3390/foods9030372 (PMC7143002; doi:10.3390/foods9030372)
Supplement: Supplementary file 1 [file foods-09-00372-s001.pdf]

Supplemental Data

Table S1. Acidity and pH of yellow water

samples	HS30	HS45	HS60
pH	3.60±0.005	3.55±0.008	3.44±0.005
acidity(mL Standard NaOH solution)	6.07±0.062	5.90±0.093	6.70±0.061

All data are presented as means±standard deviations (n=3). HS30: yellow water at 30th day, HS45: yellow water at 45th day, HS60: yellow water at 60th day. Standard NaOH solution: 0.1 mol/L.

Table S2. Aroma components of yellow water and pit mud

Aroma	HS30	HS45	HS60	JN
acetic acid(AA)(mg/L)	20.01±1.46	124.61±12.64	261.78±10.64	320.58±10.28
butyric acid(BA) (mg/L)	27.38±1.68	65.54±4.98	126.89±4.57	510.24±14.22
caproic acid(CA) (mg/L)	564.33±22.52	607.50±21.29	960.01±47.88	5050.93±402.30
ethyl acetate(EA) (mg/L)	25.44±1.12	26.50±1.01	37.85±2.06	35.63±1.22
ethyl lactate(EL) (mg/L)	25.43±0.81	82.73±3.44	181.16±6.58	231.38±12.98
ethyl butyrate(EB) (mg/L)	17.69±1.20	16.70±1.15	21.45±0.65	18.52±0.98
ethyl caproate(EC) (mg/L)	446.08±12.63	471.84±15.09	585.38±10.03	947.09±33.81

All data are presented as means±standard deviations (n=3), HS30: yellow water at 30th day, HS45: yellow water at 45th day, HS60: yellow water at 60th day, JN: pit mud

Table S3. Sequence number and diversity indices calculated based on a cutoff of 97% similarity

Sample	sequence number	Shannon	Chao1
HS30_1	39550.00	1.31	345.78
HS30_2	31572.00	1.26	389.11
HS30_3	36633.00	1.36	353.55
HS45_1	30381.00	1.09	341.68
HS45_2	39559.00	2.50	413.67
HS45_3	35917.00	2.03	360.65
HS60_1	33765.00	2.93	447.07
HS60_2	42335.00	2.59	430.41
HS60_3	50601.00	2.22	378.69
JN_1	40528.00	2.62	258.90
JN_2	44369.00	3.09	308.65
JN_3	52008.00	2.86	316.77

HS30: yellow water at 30th day, HS45: yellow water at 45th day, HS60: yellow water at 60th day, JN: pit mud

Table S4. Relative abundances of microbe communities in the pit mud and yellow water

Phylum	HS30	HS45	HS60	JN
Firmicutes	97.79%	97.65%	96.48%	98.91%
Bacteroidetes	0.67%	1.06%	1.40%	0.02%

HS30: yellow water at 30th day, HS45: yellow water at 45th day, HS60: yellow water at 60th day, JN: pit mud

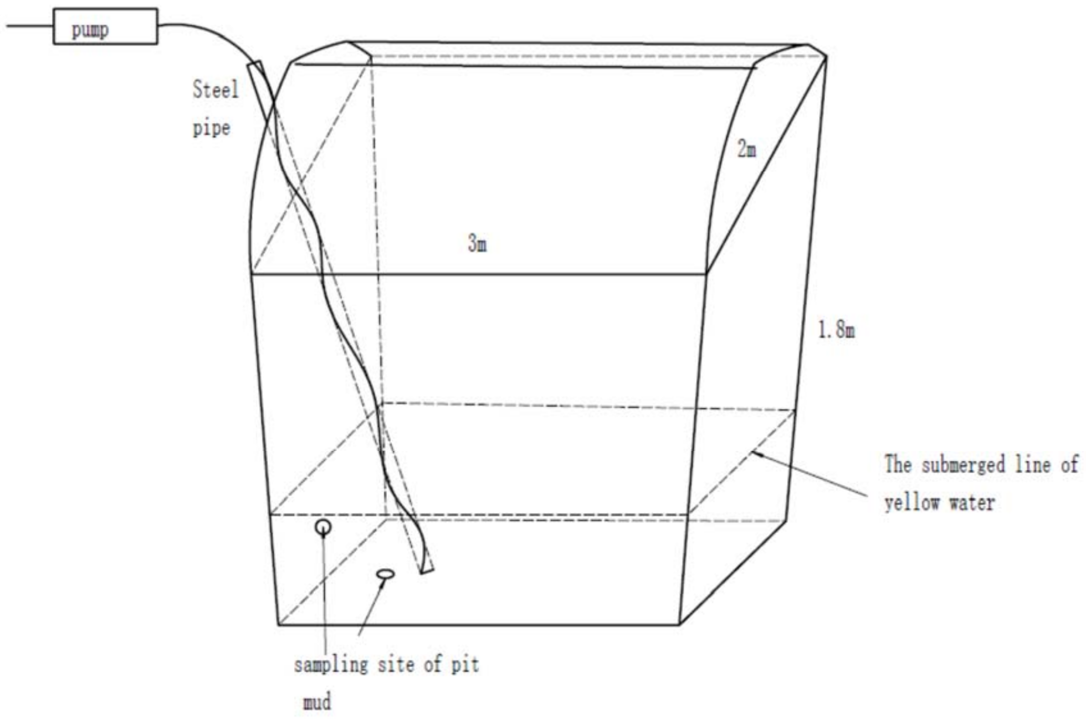

Figure S1. Sampling yellow water and the sample points of pit mud
